# Visualization of PS/γ-Secretase Activity in Living Cells

**DOI:** 10.1016/j.isci.2020.101139

**Published:** 2020-05-07

**Authors:** Masato Maesako, Nicole M. Sekula, Anna Aristarkhova, Polina Feschenko, Lauren C. Anderson, Oksana Berezovska

**Affiliations:** 1 Alzheimer's Disease Research Unit, MassGeneral Institute for Neurodegenerative Disease, Massachusetts General Hospital, Harvard Medical School, 114, 16th Street, Charlestown, MA 02129, USA

**Keywords:** Biological Sciences, Cell Biology, Methodology in Biological Sciences, Neuroscience

## Abstract

A change in Presenilin (PS)/γ-secretase activity is linked to essential biological events as well as to the progression of many diseases. However, not much is known about how PS/γ-secretase activity is spatiotemporally regulated in cells. One of the limitations is lack of tools to directly monitor dynamic behavior of the PS/γ-secretase in intact/live cells. Here we present successful development and validation of the Förster resonance energy transfer (FRET)-based biosensors that enable quantitative monitoring of endogenous PS/γ-secretase activity in live cells longitudinally on a cell-by-cell basis. Using these FRET biosensors, we uncovered that PS/γ-secretase activity is heterogeneously regulated among live neurons.

## Highlights

• C99 R-G biosensor reports endogenous PS/γ-secretase activity in live cells
• Modified donor/acceptor and linker length result in higher sensitivity C99 Y-T sensor(s)
• APP C99 in the C99 Y-T biosensor can be replaced with Notch1 N100
• PS/γ-secretase activity is heterogeneously regulated among live neurons

## Graphical Abstract

**Figure:**
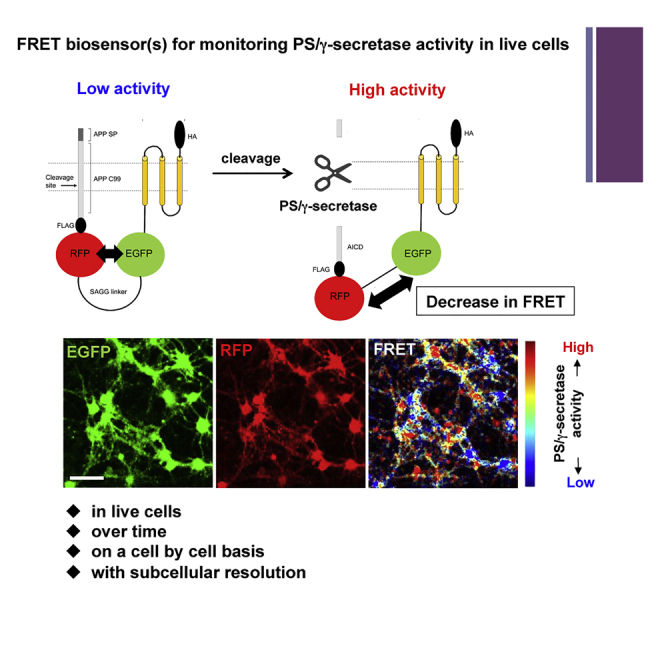

## Introduction

Presenilin (PS)/γ-secretase is a membrane-embedded aspartic protease responsible for the proteolytic processing of a wide variety of membrane-associated proteins that include the amyloid precursor protein (APP) and Notch1 (De Strooper et al., 1998, De Strooper et al., 1999, Wolfe et al., 1999). The proteolytic cleavage of APP by BACE1/β-secretase releases the APP extracellular domain and generates the membrane-bound APP C99 fragment that is an immediate substrate of PS/γ-secretase. The first step cleavage of APP C99 by PS/γ-secretase, which is known as epsilon-cleavage, generates longer Aβ48 or Aβ49 peptides and the APP intracellular domain (AICD). Longer Aβ peptides are subsequently processed by PS/γ-secretase (so-called gamma cleavages) to produce shorter Aβ37- Aβ43 peptides (Qi-Takahara et al., 2005). The accumulation in the brain of Aβ peptides, Aβ42 in particular, is one of the hallmarks of Alzheimer's disease (AD). In the case of Notch1, the N-terminally truncated Notch1 by a furin-like protease and ADAMs/α-secretase is further processed by PS/γ-secretase. This PS/γ-secretase-mediated Notch1 processing generates Notch intracellular domain (NICD), resulting in transcriptional changes in the nucleus, which is known as Notch signaling (Schroeter et al., 1998).

PS/γ-secretase is widely expressed throughout the body and plays a pivotal role in essential biological events during development, e.g., neurogenesis and skeletal formation (Shen et al., 1997, Wong et al., 1997). It has been also suggested that changes in PS/γ-secretase activity are linked to the pathogenesis of numerous diseases that are related to brain, skin, immune system, etc. (reviewed in Jurisch-Yaksi et al., 2013). Yet, little is known about how PS/γ-secretase activity is spatiotemporally regulated, as there are no tools currently available to “visualize” PS/γ-secretase activity in living cells. A few PS/γ-secretase activity assays, including the cell-free *in vitro* activity assay (Kakuda et al., 2006) or the cell-based reporter assay (Cao and Sudhof, 2001, Hansson et al., 2006, Ilagan et al., 2011) have been developed; however, shortcomings in these assays do not permit investigation of the dynamics of endogenous PS/γ-secretase activity on a cell-by-cell basis and over time. Here we report successful development, optimization, and validation of the Förster resonance energy transfer (FRET)-based biosensors for monitoring the activity of endogenous PS/γ-secretase in intact and/or live cells. These biosensors allow direct visualization of PS/γ-secretase within an individual neuron and demonstrate that PS/γ-secretase activity is differently regulated in various neurons over time.

## Results

### Development of the C99 R-G Biosensor

**Figure 1:**
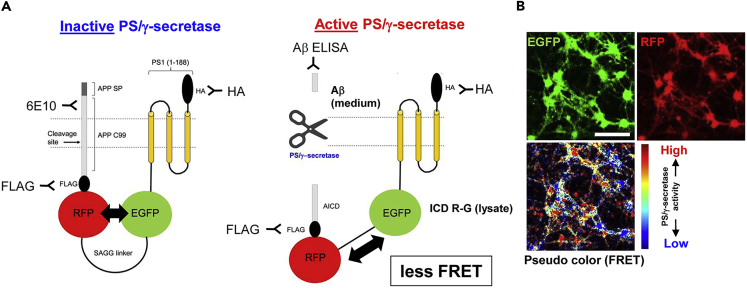
Development of the C99 R-G Biosensor (A) Schematic representation of the C99 R-G FRET biosensor. Endogenous PS/γ-secretase cleaves the APP C99, which results in the production of Aβ peptides and the APP intracellular domain (ICD) R-G, and causes a low FRET efficiency between the EGFP and the RFP. (B) The AAV-mediated expression of C99 R-G probe in mouse cortex primary neurons verified by confocal microscopy, and the pseudo-colored image corresponding to FRET efficiency measured by spectral FRET analysis. Scale bar, 100 μm.

PS/γ-secretase is responsible for the processing of a wide variety of membrane associated proteins and thus potentially influences numerous cellular pathways. However, there are no tools or assays currently available to monitor dynamic changes in the activity of PS/γ-secretase in live cells over time on a cell-by-cell basis and ultimately to help identify molecular regulators of its activity. This study aims to develop a biosensor that would enable exploring the dynamic nature of PS/γ-secretase. The C99 RFP-EGFP (C99 R-G) biosensor is an ideal molecular probe for the FRET-based assay, since in addition to an immediate PS/γ-secretase substrate, APP C99, it contains two fluorescent proteins, EGFP (donor) and RFP (acceptor) expressed in a 1:1 ratio (Figure 1A left and 1B). The C terminus of human APP C99 is tagged with RFP and EGFP is connected to the RFP with 20 amino acids (a.a.) SAGG-repeat linker (Komatsu et al., 2011). To increase FRET detection capability, the EGFP is stabilized near the membrane by fusion to the N-terminal portion of PS1. This anchor domain contains 1–188 a.a. spanning only the N terminus and first three transmembrane domains of PS1. Of note, this fragment does not contain any known binding sites for co-factors (nicastrin, Aph1, and Pen2) (Kaether et al., 2004, Watanabe et al., 2005) or the catalytic core (Wolfe et al., 1999) of PS/γ-secretase and thus does not possess functional PS/γ-secretase activity. For selective biochemical detection of the C99 R-G processing, FLAG and HA tags are inserted at the C terminus of APP C99 and after the PS1 a.a. 188, respectively. The cleavage of APP C99 within the C99 R-G biosensor by endogenous PS/γ-secretase releases Aβ peptides and APP intracellular domain (ICD) R-G (Figure 1A right). This results in a change in the proximity and/or orientation between the EGFP and the RFP, which we record by ratiometric spectral FRET analysis (Uemura et al., 2009, Maesako et al., 2017) as a reduction in the FRET efficiency. The value of the FRET efficiency can be color coded and mapped over the entire image of a cell. Therefore, the measurement of FRET efficiency permits the “visualization” of PS/γ-secretase-mediated APP C99 processing within a cell (Figure 1B).

### The C99 R-G Biosensor Is Cleaved by PS/γ-Secretase

**Figure 2:**
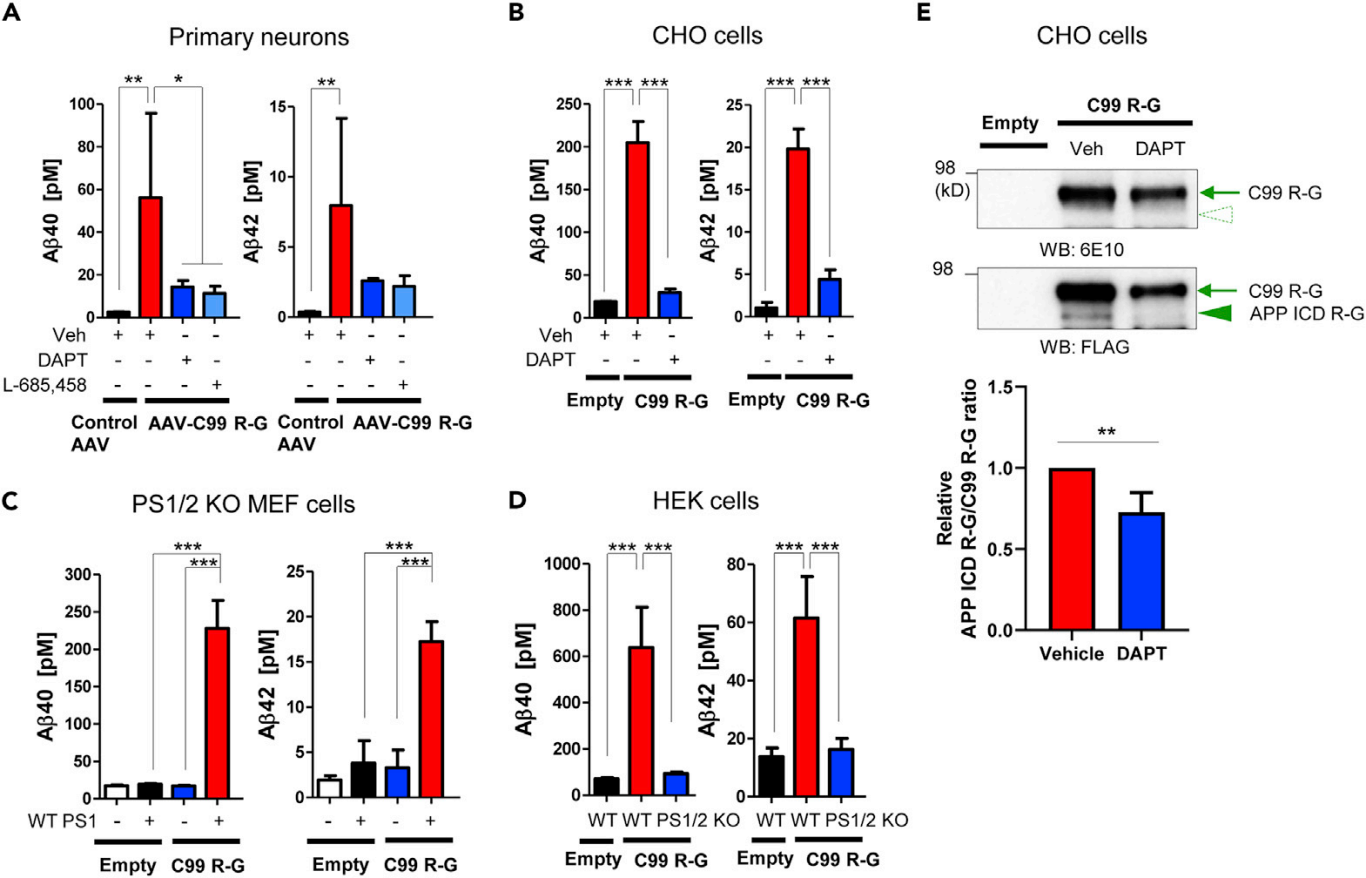
The C99 R-G Biosensor Is Cleaved by PS/γ-Secretase Detection of human Aβ40 and Aβ42 in the conditioned medium of C99 R-G expressing primary neurons (A), CHO (B), PS1/2 KO MEF + WT PS1 (C), or HEK cells (D). Aβ generation was inhibited by the treatment with PS/γ-secretase inhibitor(s) or the knockdown of PS1/2 expression. n = 4–5 biological replicates, mean ± SD, ∗p < 0.05, ∗∗p < 0.01, ∗∗∗p < 0.001, one-way factorial ANOVA. (E) Western blotting using Novex 6% Tris-Glycine gels reveals APP ICD R-G band (arrowhead) detected with the FLAG antibody but not with the 6E10 antibody in CHO cell lysates. The arrow indicates full-length C99 R-G. The ratio of APP ICD R-G band over C99 R-G was quantified and the relative ratio was shown (vehicle is set as 1). n = 3 biological replicates, mean ± SD, ∗∗p < 0.01, one-sample t test.

Cell fractionation revealed that the C99 R-G probe is integrated into the membrane (Figure S1A). Furthermore, the cell surface expression of C99 R-G was verified by a biotinylation assay confirming that the C99 R-G biosensor is trafficked through the secretory pathway (Figure S1B). To further ensure that the C99 R-G biosensor is successfully cleaved by endogenous PS/γ-secretase, we measured the level of Aβ in the conditioned medium using human Aβ40 and Aβ42 ELISA. The expression of C99 R-G in primary neurons as well as in CHO cells allowed for a clear detection of human Aβ40 and Aβ42 in the conditioned medium at approximately 10:1 ratio of the Aβ40 to Aβ42. The Aβ generation was inhibited by the treatment with PS/γ-secretase inhibitor(s), DAPT or L-685,458, verifying the specificity of the detection (Figures 2A and 2B). Human Aβ40 and Aβ42 were also detected in the conditioned media of genetically manipulated MEF and HEK cells expressing C99 R-G and PS1 (Figures 2C and 2D). In addition, western blotting was performed to detect the APP intracellular domain R-G (APP ICD R-G) in the lysate of C99 R-G expressing cells. The 6E10 antibody binding to an epitope on the N terminus of C99 R-G detected a single band around 95 kD corresponding to C99 R-G in the lysates of both vehicle and DAPT-treated cell. On the other hand, the FLAG antibody detected a lower-molecular-weight band corresponding to APP ICD R-G, which was significantly diminished by the DAPT treatment (Figure 2E). To examine the stability of C99 R-G and APP ICD R-G, the cycloheximide chase assay was performed. We found that the half-life of C99 R-G and APP ICD R-G is approximately 4 h (Figure S1C), which is significantly longer than that of endogenous C99 and APP ICD (Dyrks et al., 1993, Kimberly et al., 2001). This suggests that the fusion with RFP-linker-EGFP-PS1 (1–188)-HA might stabilize the C99 and APP ICD. Overall, these results verify that the C99 R-G biosensor is cleaved by endogenous PS/γ-secretase.

### PS/γ-Secretase-Mediated Processing of the C99 R-G Biosensor Causes Decrease in FRET between the EGFP and the RFP

Next, we used the ratiometric spectral FRET assay we set up in the laboratory (Uemura et al., 2009, Maesako et al., 2017) to determine if the FRET signal between the EGFP and RFP fluorophores within the C99 R-G probe would change after endogenously expressed PS/γ-secretase cleaves the probe. In the spectral FRET assay, the EGFP (donor) within C99 R-G is selectively excited by an Argon laser at 488 nm and emitted fluorescence is simultaneously detected by eight channels within the 456- to 617-nm wavelength range (21.4 nm bandwidth). The ratio of the fluorescence emission intensity in the 596 ± 10.7-nm channel (emission peak of RFP) to the emission in 510 ± 10.7-nm channel (emission peak of EGFP) yields the 596(R)/510(G) ratio that is used as readout of the FRET efficiency. The lower 596(R)/510(G) ratio denotes lower FRET efficiency, and in the case of C99 R-G biosensor, would indicate higher PS/γ-secretase activity.

**Figure 3:**
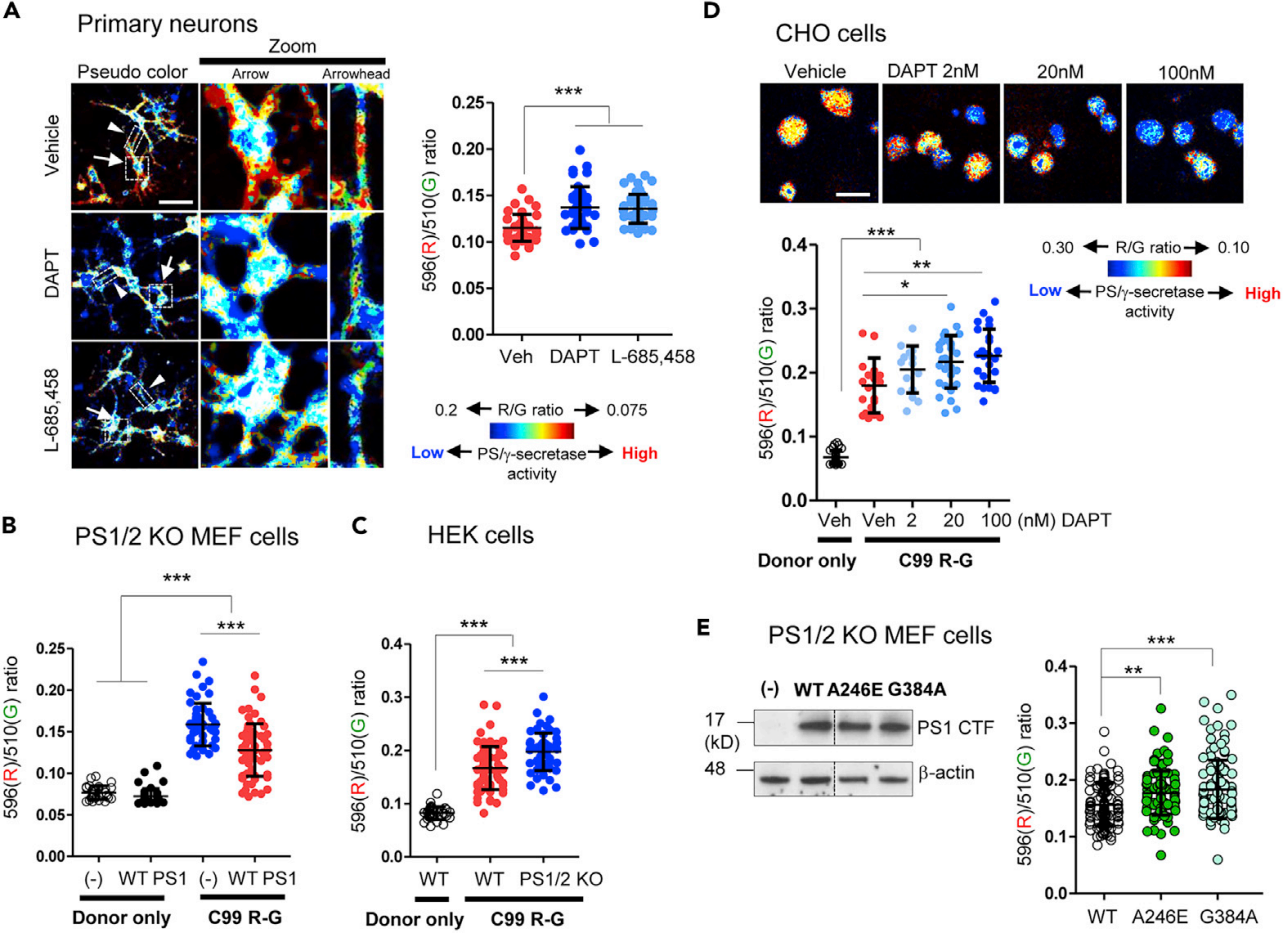
PS/γ-Secretase-Mediated Processing of the C99 R-G Biosensor Causes Decrease in FRET between the EGFP and the RFP (A) The pseudo-colored images of the 596(R)/510(G) ratio in primary neurons infected with the AAV-C99 R-G. The square and rectangle in low-magnification images (left) pointed by arrowhead and arrow, respectively, correspond to the high magnification images (middle and right). The color-coded images were generated by MATLAB using 0.075–0.2 range of the 596(R)/510(G) ratios to visually amplify the difference between experimental conditions. The overall 596(R)/510(G) ratio in processes of neurons treated with DAPT or L-685,458 (1 μM,16 h) is significantly higher than that in vehicle control (n = 40–47 regions of interest [ROIs] on neuronal processes), as shown by predominantly blue pixels. Scale bar, 50 μm. (B) The stable expression of WT PS1 in the PS1/2 KO MEF cells, i.e., re-constitution of the γ-secretase activity, results in the lower 596/510 ratio (n = 31–64 cells). (C) The knockdown of PS1/2 in HEK cells significantly increases the 596(R)/510(G) ratio (n = 31–73 cells). (D) Dose-dependent increase in the 596(R)/510(G) ratio in CHO cells treated with DAPT for 16 h (n = 16–30 cells). The pseudo-colored images suggest heterogeneous inhibition of γ-secretase activity. Scale bar, 20 μm. (E) Comparable expression levels of the active PS1 in the PS1/2 KO MEF cells stably expressing WT, A246E, or G384A PS1 (top panel). The 596(R)/510(G) ratio in the PS1/2 KO MEF + A246E PS1 or G384A PS1 cells was significantly higher than that in the PS1/2 KO MEF + WT PS1 cells (n = 102–115 cells) (bottom). Mean ± SD, ∗p < 0.05, ∗∗p < 0.01, ∗∗∗p < 0.001, one-way factorial ANOVA.

First, we verified the cleavage of the C99 R-G biosensor by PS/γ-secretase indeed results in reduced FRET efficiency. For this, mouse primary neurons expressing the C99 R-G biosensor were treated with DAPT or L-685,458 (PS/γ-secretase inhibitors, GSIs) to block the activity of endogenous PS/γ-secretase. The overall 596(R)/510(G) ratio in DAPT- or L-685,458-treated neurons was significantly higher than that in the vehicle-treated cells, indicating that high FRET efficiency is associated with low PS/γ-secretase activity (Figure 3A). The pseudo-colored image allows one to visualize regional differences in the FRET efficiency in vehicle-treated cells, revealing that PS/γ-secretase activity varies at the cell periphery versus perinuclear area or in different loci within the processes (Figure 3A). To corroborate the finding in different cell types and with a complementary genetic approach, we measured FRET efficiency in C99 R-G biosensor expressing MEF cells lacking both PS1 and PS2 (PS1/2 KO). The rescue of PS/γ-secretase activity by the stable expression of wild-type (WT) PS1 resulted in lowering of the 596(R)/510(G) ratio (Figure 3B). Similarly, the 596(R)/510(G) ratio was significantly lower in C99 R-G-expressing WT HEK cells, as compared with HEK cells in which PS1/2 expression is suppressed by the CRISPR-Cas9. These results are consistent with the higher 596(R)/510(G) ratio in cells representing reduced (lacking) PS/γ-secretase activity (Figure 3C).

To determine the ability of the C99 R-G biosensor to report progressing changes in the PS/γ-secretase activity, as oppose to “on”/“off” activity recording, the CHO cells expressing C99 R-G probe were treated with DAPT (IC50 = 20 nM) at different concentrations. The spectral FRET imaging revealed a dose-dependent increase in the 596(R)/510(G) ratio with increasing DAPT concentrations, demonstrating that the C99 R-G sensor can report gradual changes in the PS/γ-secretase activity (Figure 3D). To ensure that differences in the 596(R)/510(G) FRET ratio do not come from a different expression level of the C99 R-G probe, we performed a correlation analysis between the 596(R)/510(G) ratio and the EGFP donor fluorescence emission intensity, which reflects the expression level of C99 R-G sensor in each cell. We found that there was no positive correlation between the 596(R)/510(G) ratio and the expression level of C99 R-G biosensor in primary neurons, CHO cells or the PS1/2 DKO MEF cells stably expressing WT PS1 (Figure S2A-C). This indicates that the difference in the FRET ratio is not dependent on the level of substrate but rather on the change in proximity and/or orientation between EGFP and RFP due to changes in the PS/γ-secretase activity.

To further validate the C99 R-G spectral FRET assay, we evaluated the relative proximity between the EGFP and RFP fluorophores in vehicle/GSIs-treated cells using a complementary FRET assay: fluorescence lifetime imaging microscopy (FLIM). FLIM monitors lifetimes of the donor fluorophore (EGFP in this case) as a measure of the proximity to an acceptor (RFP) fluorophore, with shorter lifetimes reflective of a closer proximity between the donor and acceptor (Berezovska et al., 2003). In cells expressing EGFP donor fluorophore only, the EGFP lifetime is significantly longer compared with that in the cells expressing C99 R-G that contain both donor and acceptor fluorophores (Figure S2D). The shortened EGFP lifetime in DAPT- or L-685,458-treated cells as compared with the vehicle control indicates closer EGFP-RFP proximity when PS/γ-secretase activity is inhibited and the probe is not cleaved (Figure S2D). This is consistent with the findings from the spectral FRET analysis.

Recent studies suggest that the majority of familial AD (FAD) mutations in PS1 cause a decrease or loss of PS/γ-secretase activity (Quintero-Monzon et al., 2011, Chávez-Gutiérrez et al., 2012, Sun et al., 2017). To test whether the C99 R-G probe would be processed differently by the WT and FAD mutant PS1/γ-secretases, we expressed the C99 R-G biosensor in PS1/2 dKO MEF cells stably transfected with WT, A246E, or G384A PS1. Western blotting revealed comparable level of PS1 expression between the WT and mutant PS1 cell lines (Figure 3E top). The spectral FRET analysis, however, showed that the 596(R)/510(G) ratio was significantly higher in the A246E- or G384A PS1-expressing MEF cells compared with that in the WT PS1 cells (Figure 3E bottom), reflecting reduced overall activity of the PS/γ-secretase in FAD mutants. Thus, the C99 R-G biosensor can reliably document the reduced PS/γ-secretase activity due to FAD PS1 mutations in intact cells.

### Optimization of the C99 R-G Biosensor

**Figure 4:**
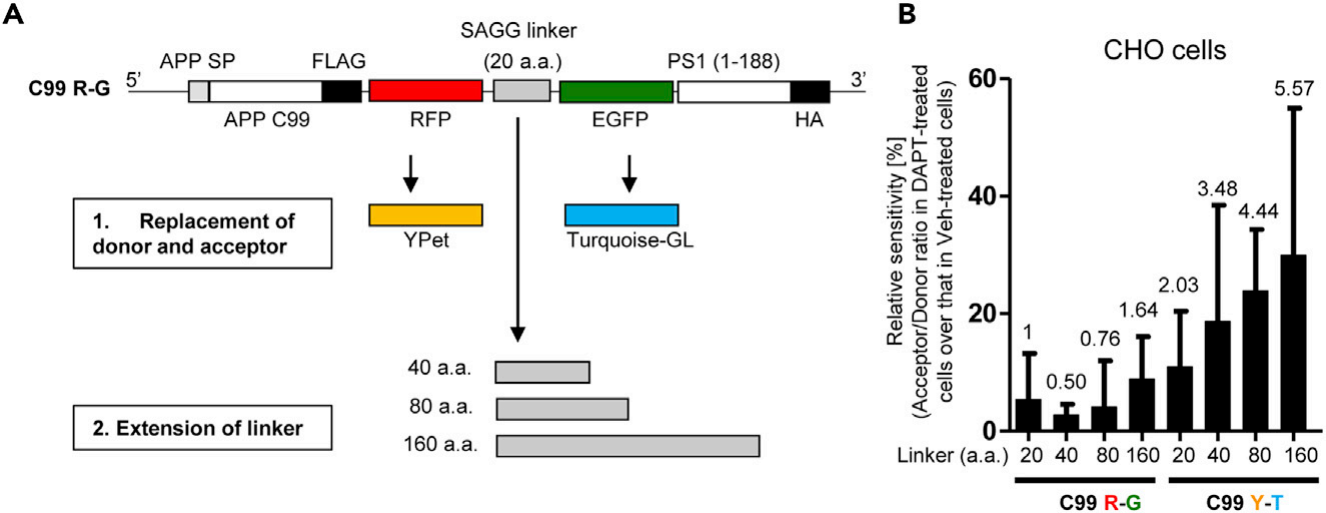
Optimization of the C99 R-G Biosensor (A) Schematic representation of the sequence of C99 R-G and C99 Y-T FRET biosensors. The EGFP (donor) and RFP (acceptor) in the C99 R-G biosensor were replaced with Turquoise-GL and YPet, respectively. The 20-a.a. SAGG linker in the C99 R-G or C99 Y-T biosensor was extended to 40, 80, or 160 a.a. (B) CHO cells expressing different biosensors were treated with 1 μM DAPT or vehicle control for 16 h. The 596(R)/510(G) ratio or 531(Y)/489(T) ratio in cells treated with DAPT was divided by that in corresponding vehicle-treated cells, and the value was multiplied by 100 to calculate the relative sensitivity of each biosensor (%) (n = 76–149 cells). The fold-change compared with the original C99 R-G (20 a.a.) biosensor (set as 1) is shown. Mean ± SD.

It has been previously reported that replacement of the donor/acceptor fluorescent proteins and extension of the linker length significantly improves the sensitivity of FRET biosensor monitoring kinases activity such as PKA or ERK (Komatsu et al., 2011). Thus, we next tested whether replacing the EGFP and RFP in the C99 R-G biosensor with Turquoise-GL (T) and YPet (Y) as a new donor and acceptor pair and/or extending the 20 a.a. SAGG linker to 40, 80, or 160 a.a. would enhance the sensitivity of the original C99 R-G biosensor (Figure 4A). The original C99 R-G (20 a.a.) biosensor and the optimized biosensors were expressed in CHO cells. The cells were treated with DAPT or vehicle control, and the 596(R)/510(G) or the 531(Y)/489(T) FRET ratio was measured by the spectral FRET analysis. To compare the sensitivity of the optimized FRET probes with the original C99 R-G (20 a.a.) biosensor, we calculated relative sensitivity of each biosensor. For this, the R/G or Y/T FRET ratio in cells expressing each probe and treated with DAPT was divided by the FRET ratio in the corresponding vehicle-treated cells. We found that replacement of the donor/acceptor fluorescent proteins and the extension of the linker length in C99 Y-T probe significantly increased the sensitivity of the original C99 R-G biosensor (Figure 4B). The cytotoxicity assay showed no significant toxicity owing to biosensor expression in CHO cells (Figure S3A). Of note, we found much lower transfection efficiency for the C99 Y-T (160 a.a.) construct compared with the other biosensors (data not shown). The C99 Y-T (80 a.a.) biosensor permits one to identify “the exceptional cells” in vehicle-treated primary neurons that show extremely high or low PS/γ-secretase activity (Figure S3B). This previously lacking capability to distinguish individual WT neurons with active and inactive PS/γ-secretase would help to understand the consequences of changes in PS/γ-secretase activity over time.

To determine whether fusion of the donor/acceptor florescent proteins and the PS1 (1–188) affects the trafficking of C99 and thus cleavage by PS/γ-secretase, we evaluated the processing efficiency between the cells expressing C99 FLAG and the C99 Y-T (80 a.a.) probe. For this we compared the ratio of the cleavage product (Aβ40 in the medium) with the level of the substrate in respective cell lysate. We found that there was no significant difference in the product/substrate ratio between the C99 FLAG- and the C99 Y-T (80 a.a.)-expressing CHO cells (Figure S3C). Thus, we conclude that fusion of the donor/acceptor florescent proteins and the PS1 (1–188) does not significantly affect the cleavage efficiency of C99 by PS/γ-secretase.

### Development and Validation of the Notch1-Based N100 Y-T Biosensor

**Figure 5:**
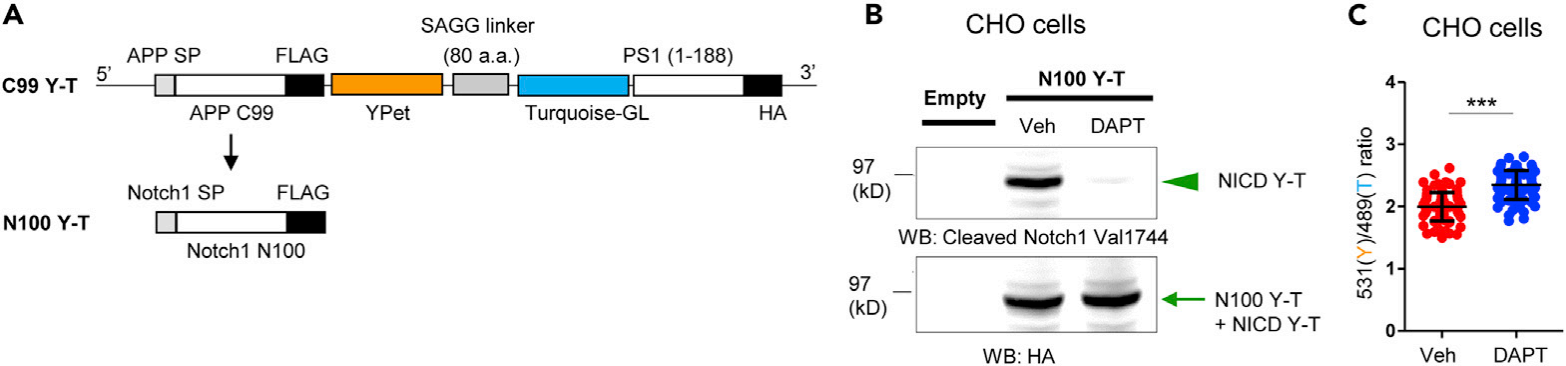
Development and Validation of the Notch1-based N100 Y-T Biosensor (A) Schematic representation of the sequence of N100 Y-T biosensor. The APP C99 in the C99 Y-T (80 a.a.) biosensor was replaced with Notch1 N100. (B) Combined full-length N100 Y-T and NICD Y-T bands are detected with the HA antibody in Western blotting using NuPAGE 4%–12% Bis-Tris Protein gels. The NICD-specific cleaved Notch1 Val1744 antibody reveals cleaved NICD Y-T band only, which is diminished by the treatment with DAPT. (C) The overall 531(Y)/489(T) ratio in DAPT-treated CHO cells expressing the N100 Y-T biosensor is significantly higher than that in vehicle control (n = 78–81 cells). Mean ± SD, ∗∗∗p < 0.001, Student's t test.

To further verify the assay is not “APP C99-specific,” we have generated a Notch1-substrate-based PS/γ-secretase activity biosensor, N100 Y-T, by replacing the APP C99 sequence in the C99 Y-T (80 a.a.) biosensor with the N- and C-terminally truncated Notch1 (N100), which is known to be an immediate substrate of PS/γ-secretase (Esler et al., 2002) (Figure 5A). In western blotting, the HA antibody reveals both N100 Y-T and NICD Y-T bands and thus shows a mixture of these two bands in vehicle-treated lysates, and mainly N100 Y-T in DAPT condition, whereas the Notch intracellular domain (NICD) antibody selectively detects only NICD Y-T band present in the lysate of vehicle treated CHO cells, but not DAPT, verifying that the N100 Y-T biosensor is processed by endogenous PS/γ-secretase (Figure 5B). Spectral FRET analysis reveals that DAPT significantly increases the 531(Y)/489(T) ratio compared with vehicle control in N100 Y-T expressing cells, showing that the cleavage of N100 Y-T biosensor by endogenous PS/γ-secretase reduces FRET efficiency between the Turquoise-GL donor and YPet acceptor (Figure 5C). Thus, similarly to the APP C99-based biosensors, the Notch1 N100 Y-T probe is successfully cleaved by and therefore could be used to report activity of the endogenous PS/γ-secretase.

### Heterogeneous Regulation of PS/γ-Secretase Activity among Different Neurons

**Figure 6:**
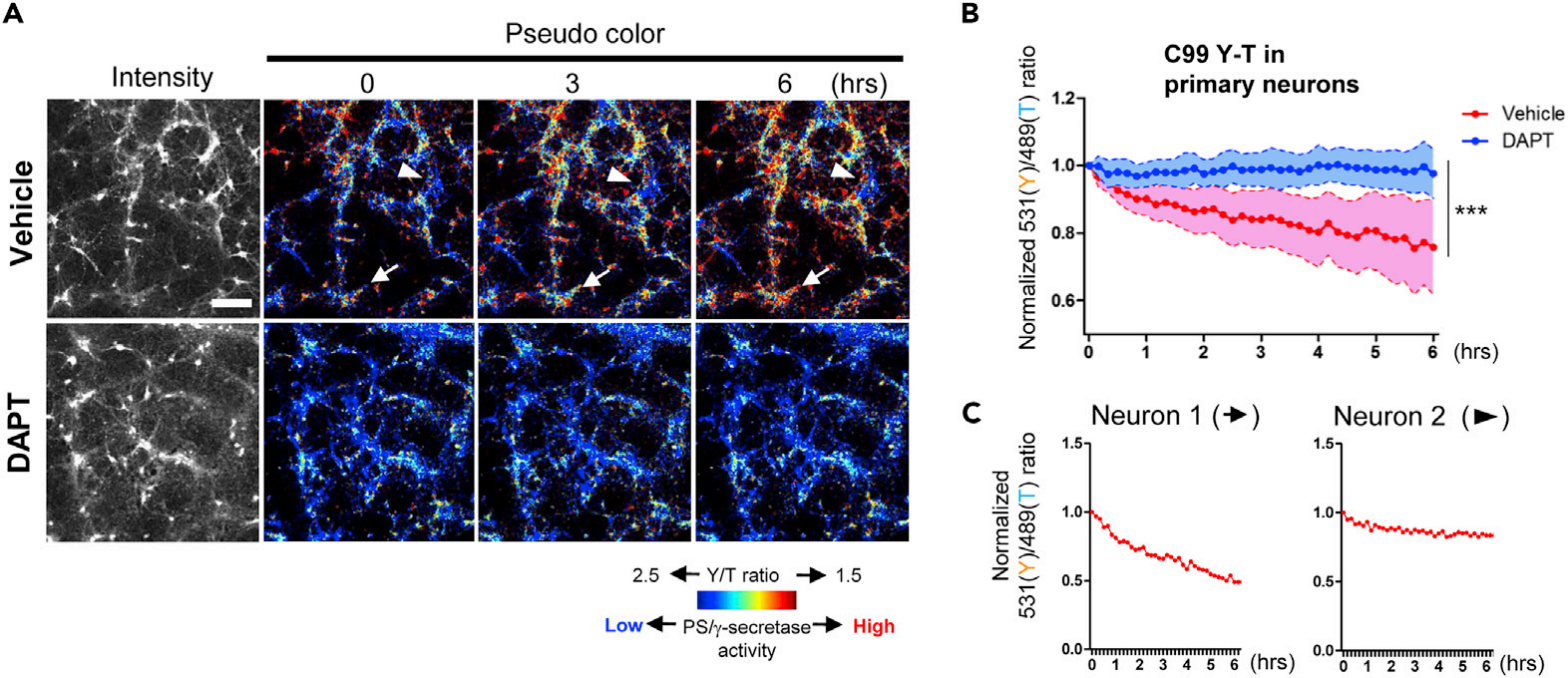
Heterogeneous Regulation of PS/γ-Secretase Activity among Different Neurons (A) The pseudo-colored images of longitudinal (for 6 h) imaging of the 531(Y)/489(T) ratio in live primary neurons infected with AAV-C99 Y-T. (B) Time-dependent decrease in the 531(Y)/489(T) ratio observed in vehicle-treated neurons is abolished by the treatment with DAPT (n = 39 cells). Mean ± SD, ∗∗∗p < 0.001, Repeated Measures ANOVA, Scale bar, 100 μm. (C) Cell-by-cell analysis reveals neurons with active (Neuron 1 highlighted by an arrow in Figure 6A) or less active PS/γ-secretase (Neuron 2 by an arrowhead).

Finally, to examine if the C99 Y-T biosensor could monitor dynamic longitudinal changes in PS/γ-secretase activity within a single cell, and thus to elucidate whether PS/γ-secretase activity is temporally and/or differentially regulated among neurons, the 531(Y)/489(T) ratio was longitudinally monitored for 6 h in live neurons treated with DAPT or vehicle control in the presence of cycloheximide. As expected, there was no change in the 531(Y)/489(T) ratio over time in neuronal cultures where the PS/γ-secretase activity was inhibited by DAPT (Figures 6A and 6B). However, we observed a gradual decrease in the overall 531(Y)/489(T) ratio in the vehicle control-treated neurons (Figures 6A and 6B), indicating that the C99 Y-T biosensor is being cleaved by PS/γ-secretase over time. More importantly, cell-by-cell analysis of the 531(Y)/489(T) ratio permitted dissociation of neuronal populations in which PS/γ-secretase would be active or inactive (Figure 6C), highlighting the heterogeneous regulation in PS/γ-secretase-mediated C99 Y-T biosensor processing among different neurons. Altogether, these results provide strong evidence that cleavage of the C99/N100 donor-acceptor biosensor(s) by endogenous PS/γ-secretase results in reduced FRET efficiency between the donor (EGFP/Turquoise-GL) and the acceptor (RFP/YPet), and thus they could be used as reliable reporters of the activity of endogenous PS/γ-secretase in its native environment in intact/live cells. Furthermore, the FRET-based biosensors we developed allowed us to uncover the dynamic and heterogeneous nature of the PS/γ-secretase in different neurons.

## Discussion

PS knockout mice revealed that PS/γ-secretase is required for normal neurogenesis, neuronal survival, and skeletal and vasculature formation in embryos, indicating that PS/γ-secretase plays a significant role during development (Shen et al., 1997, Wong et al., 1997). Approximately 100 different substrates of PS/γ-secretase have been identified (Haapasalo and Kovacs, 2011), stressing that changes in the activity of PS/γ-secretase would potentially affect numerous cell pathways. Indeed, conditional knockout of the PS1/2, Nicastrin, or Aph1 in the excitatory neurons of adult mouse brain reveals age-dependent neuronal loss (Saura et al., 2004; Tabuchi et al., 2009; Acx et al., 2017), supporting a crucial role of PS/γ-secretase in neuronal survival. Furthermore, over 300 missense mutations that cause early onset of AD have been identified on the genes encoding PS1 and PS2 (http://www.alzforum.org/mutations), emphasizing the importance of PS/γ-secretase in the pathogenesis of AD. Together, these highlight the significant roles of PS/γ-secretase in neuronal survival and neurodegeneration.

Several assays for monitoring PS/γ-secretase activity have been previously developed, such as the cell-free *in vitro* PS/γ-secretase activity assay (Kakuda et al., 2006), the cell-based reporter assays (Cao and Sudhof, 2001, Hansson et al., 2006, Ilagan et al., 2011), or the C99 GFP-based fluorescence membrane retention assay (Florean et al., 2008). The major advantage of the cell-free *in vitro* activity assay, which is widely used in many studies (including ours), is its high sensitivity; however, it allows neither a cell-by-cell-based analysis nor longitudinal monitoring of the PS/γ-secretase activity. Moreover, it is unclear if the activity of PS/γ-secretase *in vitro* truly reflects that in live/intact cells. On the other hand, the cell-based reporter assay (Gal4-dependent Luciferase expression, split GFP, etc.) and the fluorescence membrane retention assay could be potentially influenced by stability of the intracellular domain. Additionally, these assays have been verified in a limited number of cell types and thus may not allow monitoring PS/γ-secretase activity in neurons. To overcome these shortcomings, we present FRET-based biosensors based on APP-C99 and Notch1-N100 substrates that enable reporting of endogenous PS/γ-secretase activity over time on a cell-by-cell basis and within the physiological environment of the cell.

A broad range of evidence from studies in *C. elegans*, flies, and mice has implicated that FAD PS1/2 mutations result in the loss of proteolytic activity of PS/γ-secretase (Levitan et al., 1996, Seidner et al., 2006, Saito et al., 2011, Xia et al., 2015). Partial loss of PS/γ-secretase activity results in multiple biological events that include relative increase in the membrane-bound C-terminal fragment and decrease in the intracellular domain of PS/γ-secretase substrates, as well as relative increase in the Aβ42 over Aβ40 peptides. Although over 20 years have passed from the first discovery of missense mutations on the genes encoding PS1/PS2 (Sherrington et al., 1995, Levy-Lahad et al., 1995), it is still unclear what is the exact molecular mechanism by which PS missense mutations result in FAD. It is worth to note that patients carrying FAD-linked PS1 mutations also express one normal copy of PS1 and two normal copies of PS2 alleles, which could partially compensate for the shortcomings of the diseased allele. On the other hand, recent studies have shown that FAD-linked mutant PS1/γ-secretase interacts with the WT PS1/γ-secretase and decreases overall PS/γ-secretase activity, demonstrating the dominant negative effect of FAD-linked mutant over WT PS1/γ-secretase (Heilig et al., 2013, Zhou et al., 2017). Our novel FRET-based biosensors could help to elucidate whether overall PS/γ-secretase activity is decreased or is not changed in the “FAD (i.e., mutant PS1 is co-expressed with WT PS1 and PS2)” intact/live neurons compared with WT neurons. Furthermore, the Aβ49/48 generated by PS/γ-secretase-mediated epsilon-cleavage of APP C99 is sequentially processed by consecutive gamma-cleavages to yield the predominant Aβ species, Aβ40/42/43 (Kakuda et al., 2006). Since the APP-C99-based biosensors report more specifically the first epsilon-cleavage by PS/γ-secretase, identification of the cells that have increased epsilon cleavage could also indicate, although indirectly, the cells in which Aβ production occurs at different rates.

Disappointingly, the AD clinical trials testing the efficacy of γ-secretase inhibitors failed owing to adverse events that include cognitive worsening (Doody et al., 2013). On the other hand, γ-secretase modulators, GSMs, have been developed and their efficacy is being assessed in clinical trials. The major advantage of the GSMs is that they spare the epsilon-cleavage of APP, as well as other substrates such as Notch1, but shift the gamma-cleavages of APP toward generation of the shorter Aβ38/37. The novel biosensors we developed would not only help to test directly whether FAD mutations diminish the Notch1/APP epsilon cleavage by an endogenous PS/γ-secretase enzyme in its normal physiological environment but also, importantly, may have preclinical therapeutic application by enabling to screen for PS/γ-secretase *modulators* that spare the ICD-generating γ-secretase-dependent cleavage of the substrates, as well as for PS/γ-secretase *activators* that could restore the decreased overall PS/γ-secretase activity due to, for example, FAD mutations.

PS/γ-secretase also plays a significant role in the pathogenesis of other diseases beyond AD (reviewed in Jurisch-Yaksi et al., 2013). For example, one of the adverse events triggered by γ-secretase inhibitors in the AD trial was skin cancer (Doody et al., 2013). Moreover, mutations on the genes of γ-secretase components such as PS1, Pen2, or Nicastrin that cause γ-secretase haploinsufficiency are reported to cause familial acne inversa (Wang et al., 2010) and/or Dowing-Degos disease associated with acne inversa (Ralser et al., 2017). It has never been clarified how the inhibition of PS/γ-secretase activity causes these skin diseases. The FRET biosensors we developed could provide important spatiotemporal information about the role of PS/γ-secretase in skin biology. Since the APP C99 part in the C99 R-G/Y-T biosensors could be easily replaced by other PS/γ-secretase substrates to test their cleavability and thus their role in various cellular pathways (Figure 5), the “γ-Substrate” donor-acceptor biosensor could be applied more broadly to investigate the roles of PS/γ-secretase in multiple cellular events or diseases.

In summary, we have developed, validated, and optimized APP C99- and Notch1 N100-based FRET biosensors for monitoring PS/γ-secretase activity in intact/live cells. Using these biosensors, we showed uneven PS/γ-secretase activity in neuronal processes as well as in different compartments of an individual neurite, suggesting that the PS/γ-secretase-mediated processing of substrates is differentially regulated within a single neuron. Moreover, the longitudinal imaging in live neurons revealed that overall PS/γ-secretase activity is heterogeneously regulated among live neurons. These findings suggest the spatiotemporally heterogeneous nature of the PS/γ-secretase activity. The FRET biosensors developed in this study would not only be useful tools to identify the molecular regulators of PS/γ-secretase activity but provide previously missing capability to distinguish cell populations with active and inactive PS/γ-secretase activity in WT neurons. Such capability would allow direct examination of the mechanistic link between change in PS/γ-secretase activity and its biological consequences such as neuronal vulnerability in WT neurons in future studies.

### Limitations of the Study

The common issues related to fluorescent imaging such as potential photobleaching due to high laser power excitation could affect the readout, and thus should be controlled for. It should also be noted that the change in FRET efficiency while using the FRET biosensors could reflect changes not only in PS/γ-secretase activity but also in the amount of active PS/γ-secretase complex. Therefore, further refinement of the assay and mechanistic studies are required in the follow-up studies.

### Resource Availability

#### Lead Contact

Further information and requests for resources and reagents should be directed to and will be fulfilled by the Lead Contact, Masato Maesako (mmaesako@mgh.harvard.edu).

#### Materials Availability

Plasmids generated in this study will be made available on reasonable requests with a completed Materials Transfer Agreement.

#### Data and Code Availability

The published article includes all datasets generated during the study.

## Methods

All methods can be found in the accompanying Transparent Methods supplemental file.

## Supplemental Information

Document S1. Transparent Methods and Figures S1–S3
