# Supplementary material for: Visualization of PS/γ-Secretase Activity in Living Cells
Source: iScience. 2020 May 7;23(6):101139. doi: 10.1016/j.isci.2020.101139 (PMC7235286; doi:10.1016/j.isci.2020.101139)
Supplement: Document S1. Transparent Methods and Figures S1–S3 [file mmc1.pdf]

iScience, Volume *23*

Supplemental Information

Visualization of PS/γ-Secretase Activity in Living Cells

Masato Maesako, Nicole M. Sekula, Anna Aristarkhova, Polina Feschenko, Lauren C. Anderson, and Oksana Berezovska

Figure S1. Membrane integration, cell surface expression and stability of the C99 R-G biosensor, Related Figure 2

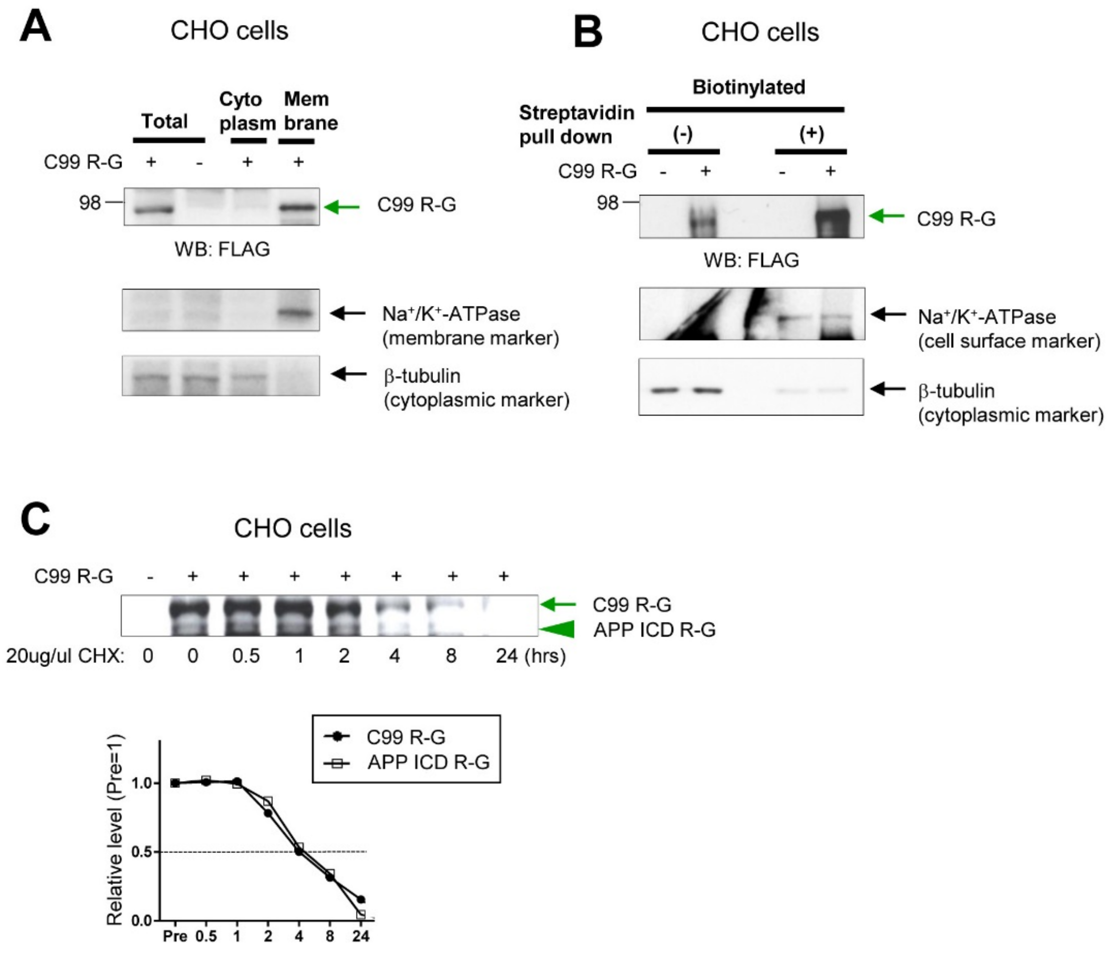

The subcellular protein fractionation (**A**) and the cell surface biotinylation (**B**) of CHO cells expressing C99 R-G biosensor. Na^+/K^+-ATPase and β-tubulin were used as membrane/cell surface markers and cytoplasmic marker, respectively. The stability of C99 R-G and APP ICD R-G was examined by the cycloheximide (CHX) chase assay (**C**).

Figure S2 Correlation analysis between the 596(R)/510(G) ratio and the expression level of biosensor, Related Figure 3

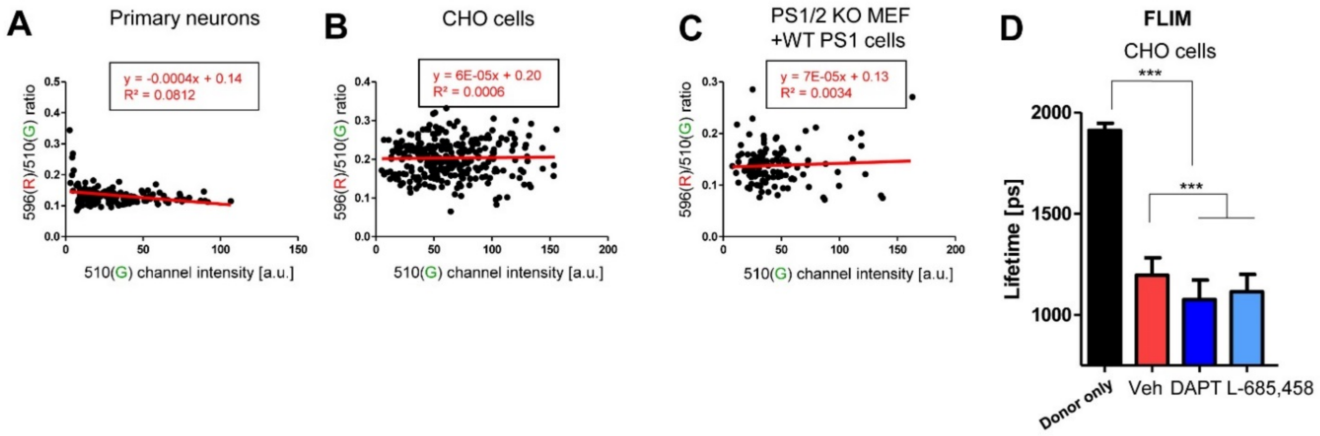

A correlation analysis between the 596(R)/510(G) ratio and the intensity in the 510(G) channel reflecting the expression level of C99 R-G biosensor in a primary neuron (**A**), CHO (**B**), or the WT PS1 stably expressing PS1/2 knockout MEF cells (**C**). FLIM analysis of the CHO cells expressing C99 R-G biosensor (**D**). The cells were treated with DAPT, L-685, 458 (1μM,16 hrs) or vehicle control. γ-Secretase inhibitors treatment resulted in the shorter lifetime of the donor EGFP than vehicle. n=29-33, Mean ± SD, ***$p < 0.001$, one-way factorial ANOVA

Figure S3 Characterization of higher sensitivity C99 Y-T biosensor(s), Related Figure 4

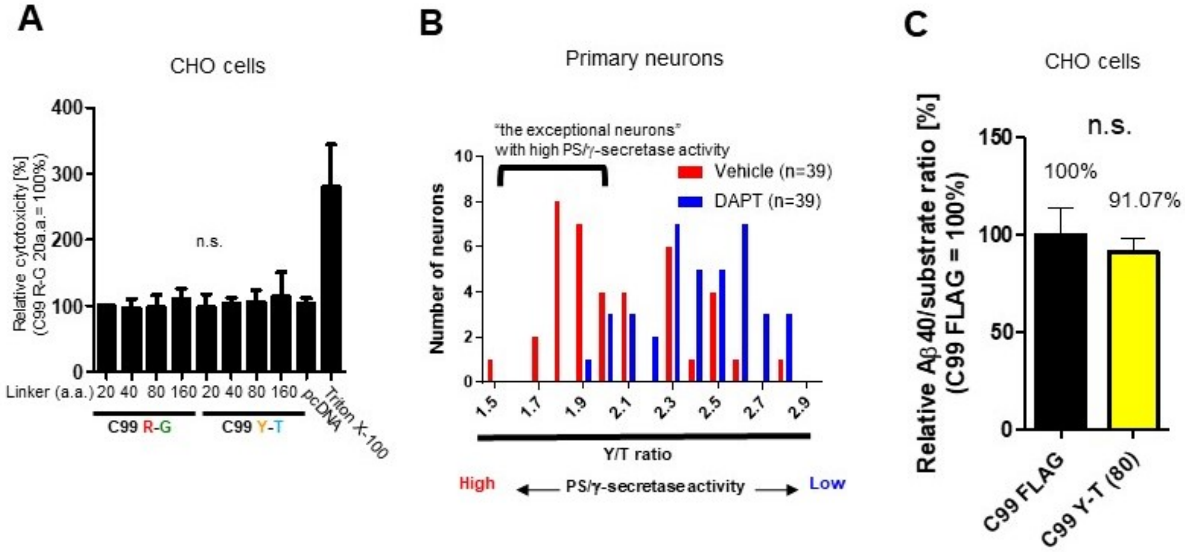

(**A**) Cytotoxicity assay in CHO cells expressing different biosensors as compared to the cells expressing control pcDNA. TritonX-100 was used as a positive control of the cytotoxicity. n=3, one-way factorial ANOVA. (**B**) Histogram of the Y/T ratio in primary neurons expressing the C99 Y-T (80a.a.) biosensor treated with DAPT or vehicle control, highlighting "the exceptional neurons" with high or low activity of PS/γ-secretase in primary neurons treated with vehicle control. (**C**) The processing efficiency between C99 and C99 Y-T (80 a.a.) biosensor. C99 FLAG or the C99 Y-T (80 a.a.) probe was expressed into CHO cells and the conditioned medium and the cell lysate were collected to measure Aβ40 (product) and the expression level of C99 FLAG/C99 Y-T (80 a.a.) (substrate), respectively. The level of Aβ40 was normalized by the level of substrate. There was no difference in the Aβ40/substrate ratio between the C99 FLAG and C99 Y-T (80 a.a.). n=3, Mean ± SD, Student's t-test

Transparent Methods

1. Plasmid DNA and AAV preparation

The C99 R-G biosensor was generated as follows: polymerase chain reaction (PCR) was performed to clone the HindIII-APP signal peptide (SP)-APP C99-FLAG-KpnI cDNA fragment. The pcDNA-APPSP-C99 FLAG (Uemura et al., 2010) was used as a template and the primers;
FW: TTTTAAGCTTACCATGCTGCCCGGTTTGGCA,
RV:TTTTGGTACCCTTGTCATCGTCGTCCTTGTA,
were used for the PCR. The KpnI-RFP-EcoRI was cloned from pcDNA-EGFP-PS1-RFP (Uemura et al., 2009) with the primers;
FW: TTTTGGTACCATGGCCTCCTCCGAGGAC,
RV: TTTTGAATTCGGCGCCGGTGGAGTGGCG.
The EcoRI-20 a.a. SAGG-EcoRV was cloned by annealing of complementary pairs of oligonucleotides,
FW:AATTCAGTGCTGGTGGTAGTGCTGGTGGTAGTGCTGGTGGTAGTGCTGGTGGTAGTGCTGGTGGTGAT,
RV:ATCACCACCAGCACTACCACCAGCACTACCACCAGCACTACCACCAGCACTACCACCAGCACTG.
The oligonucleotides diluted in 10 mM Tris, 1 mM EDTA, 50 mM NaCl (pH 8.0) were boiled for 5 minutes, followed by gradually reducing the heat (-1 °C /1min) using a PCR thermocycler. The EcoRV-EGFP-PS1 1-188 a.a.-HA-XbaI was cloned from pcDNA-EGFP-PS1 (Uemura et al., 2009) with the primers;
FW: TTTTGATATCACCATGGTGAGCAAGGGCGAGGAG,
RV:TTTTTCTAGACTAAGCGTAATCTGGAACATCGTATGGGTAGGTTTTAAACACTTC.
The cloned cDNA fragments were ligated by Rapid DNA Ligation Kit (MilliporeSigma, Burlington, MA) and incorporated into pcDNA 3.1(+) vector (Thermo Fisher Scientific, Waltham, MA). To generate the donor only negative control of the FRET assay: C99 EGFP (G), the cDNA fragments without the KpnI-RFP-EcoRI were ligated.

To develop the C99 Y-T biosensors, the EcoRI restriction sites in the sequence of C99 and EGFP-PS1(1-188) in pcDNA3.1-C99 R-G were broken. The cDNA of Turquoise-GL and that of YPet were amplified from AKAR3EV (Komatsu et al., 2011) using the primer pairs:
FW: TTTTGATATCACCATGGTGAGCAAGGGCGAGGA,
RV: TTTTGGATCCCAGCTCGTCCATGCCGAGAGTGAT, and
FW: TTTTGGTACCATGTCTAAAGGTGAAGAATTATT
RV: TTTTGAATTCGTACAATTCATTCATACCCTCGG, respectively. The amplified cDNA was sub-cloned into the pcDNA3.1-C99 R-G. To extend the linker length, the EcoRI-40 a.a. SAGG-EcoRV, the EcoRI-80 a.a. SAGG-EcoRV and the EcoRI-160 a.a. SAGG-EcoRV were synthesized by GenScript (Piscataway, NJ) and sub-cloned into the pcDNA3.1-C99 R-G (20a.a.) or the pcDNA3.1-C99 Y-T (20a.a.).

For the development of N100 Y-T biosensor, the HindIII-Notch 1 SP- the extracellular deleted Notch 1(NΔE)-KpnI was first cloned from the pCS2-NΔE (Mumm et al., 2000) using the primers; FW: TTTTAAGCTTACCATGCCACGGCTCCTGACGCCC, RV: TTTTGGTACCTAGTTCTAGAACTAGTGGATCCCC, and sub cloned into the pcDNA3.1-C99 Y-T (80a.a.). Then, the NΔE was replaced with the N100 FLAG which was amplified using primers; FW: TTTTATCGATGTGAAGAGTGAGCCGGTGGAGCCT, RV:

TTTTGGTACCCTTGTCATCGTCGTCCTTGTAGTCGGTCTCCAGGTCTTCGTC. All constructs were sequenced.

To prepare the AAV which carries C99 R-G (20a.a.) or C99 Y-T (80a.a.), the cDNA was sub-cloned into a pAAV2/8 vector containing human Synapsin 1 and WPRE sequences (Maesako et al., 2017). The packaging into viruses was performed at Gene Transfer Vector Core, Massachusetts Eye and Ear Infirmary (Boston, MA) or University of Pennsylvania Gene Therapy Program vector core (Philadelphia, PA). The virus titer was 1.11E+13 GC/mL (AAV-C99 R-G) and 4.95E+13 GC/mL (AAV-C99 Y-T).

2. Cell culture

Primary neuronal cultures were obtained from cerebral cortex of mouse embryos at gestation day 14–16 (Charles River Laboratories, Wilmington, MA). The neurons were dissociated using Papain Dissociation System (Worthington Biochemical Corporation, Lakewood, NJ) and were maintained for 13-15 days in vitro (DIV) in Neurobasal medium containing 2% B27 supplement, 1% GlutaMAX Supplement, and 1% Penicillin Streptomycin (Pen Strep) (Thermo Fisher Scientific, Waltham, MA). The neuronal preparation procedure was in compliance with the NIH guidelines for the use of animals in experiments and was approved by the Massachusetts General Hospital Animal Care and Use Committee (2003N000243). PS1/PS2 double knockout mouse embryonic fibroblasts (PS1/2 KO MEF), and the WT, A246E or G384A PS1 stably expressing PS1/2 KO MEF cell lines were kind gifts from Dr. Bart De Strooper (University of Leuven, Leuven, Belgium). PS1/PS2 double knockout Human Embryonic Kidney cells (PS1/2 KO HEK) and parental WT HEK cells were kind gifts from Dr. Dennis Selkoe (Brigham and Women's Hospital, Harvard Medical School, Boston MA). Chinese hamster ovary (CHO) cells, obtained from ATCC (American Type Culture Collection, Manassas, VA), were maintained in Opti-MEM Reduced Serum Medium (Thermo Fisher Scientific, Waltham, MA) supplemented with 5% FBS (Atlanta Biologicals Inc, Flowery Branch, GA), the PS1/2 KO MEF cells were in Opti-MEM Reduced Serum Medium + 5% FBS + 1% Pen Strep with or without selection antibiotic: puromycin, 5ug/mL (Thermo Fisher Scientific, Waltham, MA), the PS1/2 KO and parental HEK cells were in DMEM (high glucose, no glutamine) + 5% FBS + 1% GlutaMAX Supplement + 1% Pen Strep. The cells were authenticated using STR profiling, monitored for mycoplasma contamination every 2 months. Lipofectamine 3000 (Thermo Fisher Scientific, Waltham, MA) was used for transient transfection according to the manufacturer's instructions.

3. Materials

Anti-β-Amyloid, 1-16 (6E10) antibody was purchased from BioLegend (San Diego, CA), anti-FLAG antibody was from FUJIFILM Wako Pure Chemical Corporation (Osaka, Japan), anti-HA antibody was from Abcam (Cambridge, MA), anti-PS1, anti-NICD (cleaved Notch1 Val1744) and anti-β-tubulin antibodies were from Cell Signaling Technology, Inc. (Danvers, MA), anti-β-actin antibody was from Sigma-Aldrich (St. Louis, MO), and anti-Na^+/K^+-ATPase antibody was from MilliporeSigma (Burlington, MA). γ-Secretase inhibitors – DAPT, L-685,458, Dimethyl sulfoxide (DMSO) vehicle control and cycloheximide were obtained from Sigma-Aldrich (St. Louis, MO).

4. Spectral FRET

An Argon laser at 488 nm or at 458 nm wavelengths was used to excite the EGFP or the Turquoise-GL in C99 R-G and C99 Y-T/N100 Y-T biosensors, respectively. The emitted fluorescence was detected by eight channels within the 456-617 nm wavelength range on a Zeiss LSM510 Meta (21.4 nm spectral bandwidth for each channel) or an Olympus FV3000 (20 nm bandwidth) confocal microscope equipped with CO2/heating units. x10/0.25 or x25/0.8 objective was used for the imaging. Average pixel fluorescence intensity for the whole cell after subtraction of the background fluorescence was measured using Image J. The ratios of fluorescence intensity in the 596 nm channel (RFP emission peak) to that in the 510 nm channel (EGFP emission peak), and the ratio of fluorescence emission in the 531 channel (emission peak of YPet) to that in the 489 channel (emission peak of Turquoise-GL) were used as readouts of the FRET efficiency, which reflect the relative proximity between the donor and the acceptor. Pseudo-colored images were generated in MATLAB (The MathWorks, Inc., Natick, MA).

5. FLIM

A mode-locked Chameleon Ti:Sapphire laser (Coherent Inc., Santa Clara, CA) set at 900 nm was used to excite the EGFP donor fluorophore. An Olympus BX50WI microscope and x20/0.75 water immersion objective was used for the imaging. The donor fluorophore lifetimes were recorded using a high-speed photomultiplier tube (MCP R3809; Hamamatsu, Bridgewater, NJ) and a time-correlated single-photon counting acquisition board (SPC-830; Becker &Hickl, Berlin, Germany). The baseline lifetime (*t*1) of the donor fluorophore was measured in the absence of the acceptor fluorophore (negative control, FRET absent). In the presence of the acceptor fluorophore (RFP), excitation of the donor fluorophore results in reduced donor emission energy if the donor and acceptor are less than 5-10 nm apart (FRET present). This yields characteristic shortening of the donor fluorophore lifetime (*t*2). The acquired FLIM data were analyzed using SPC Image software (Becker &Hickl, Berlin, Germany) by fitting the data to one (donor only negative control) or two (acceptor present) exponential decay curves. In the latter case, *t1* of the non-FRETing population was “fixed” and thus excluded from the analysis, and only shorter, *t2*, values were analyzed.

6. Aβ ELISA

The conditioned medium of cells was collected, centrifuged for 5 minutes at 600 g, and the supernatant was diluted and used to measure human Aβ40 and Aβ42 levels. The Human β Amyloid (1-40) ELISA Kit wako II and Human β Amyloid (1-42) ELISA Kit Wako were used for the measurement according to the manufacture’s protocol (FUJIFILM Wako Pure Chemical Corporation, Osaka, Japan).

7. Western blotting

The cells were lysed in a cell lysis buffer (1% Triton X-100, 0.25% NP-40, 10mM Tris, 2mM EDTA, 150mM NaCl, pH 7.4) with protease and phosphatase inhibitor cocktail (Thermo Fisher Scientific, Waltham, MA) and incubated for 30 minutes on ice. Each sample was then centrifuged, and the supernatants were collected. Protein concentrations were determined using a Pierce BCA Protein Assay Kit (Thermo Fisher Scientific, Waltham, MA). The concentration-normalized samples were diluted in Novex™ Tris-Glycine SDS Sample Buffer or NuPAGE™ LDS Sample Buffer, and NuPAGE™ Sample Reducing Agent (Thermo Fisher Scientific, Waltham, MA). After boiling, the samples were subjected to SDS-PAGE on Novex™ 6% Tris-Glycine Mini or NuPAGE™ 4-12% Bis-Tris Protein gels (Thermo Fisher Scientific, Waltham,

MA), followed by transferring to nitrocellulose membranes (Thermo Fisher Scientific, Waltham, MA) using the BioRad Wet electroblotting system (BioRad, Hercules, CA). The detection was performed by immunoblotting with specific primary and corresponding fluorophore or HRP-conjugated secondary antibodies, and developing the membranes using the digital imaging system LI-COR Odyssey CLx scanner (LI-COR Biosciences, Lincoln, NE), or Western Lightning Plus-ECL (PerkinElmer, Waltham, MA) and Amersham Hyperfilm™ ECL (GE Healthcare, Chicago, IL).

8. Subcellular protein fractionation

The cytosolic and membrane fractions from cells were purified using Subcellular Protein Fractionation Kit for Cultured Cells (Thermo Fisher Scientific, Waltham, MA) according to the manufacture's protocol. The successful purification was verified by the detection of β-tubulin (the cytosolic fraction) and Na^+/K^+-ATPase (the membrane fraction).

9. Cell surface biotinylation

Proteins expressing on the cell surface were labeled by the incubation with EZ-Link™ Sulfo-NHS-SS-Biotin (0.3 mg/mL) (Thermo Fisher Scientific, Waltham, MA) for 30 minutes on ice. After wash by PBS, the cells were lysed in RIPA buffer (Sigma-Aldrich, St. Louis, MO) with protease and phosphatase inhibitor cocktail and incubated for 30 minutes on ice. The samples were then centrifuged, the supernatants were collected, and protein concentrations were normalized using a Pierce BCA Protein Assay Kit. The biotin-labelled proteins were pulled-down by Streptavidin Magnetic Beads (New England BioLabs Inc., Ipswich, MA), and eluted in the elution buffer (32.5 mM Glycine pH 2.8, Novex™ Tris-Glycine SDS Sample Buffer (1X), NuPAGE™ Sample Reducing Agent (1X) (Thermo Fisher Scientific, Waltham, MA)). After boiling, the samples were subjected to SDS-PAGE.

10. Cycloheximide chase assay

The cells were treated with 20ug/ul cycloheximide (CHX) to inhibit new protein synthesis. Then, the cells were lysed in a cell lysis buffer (1% Triton X-100, 0.25% NP-40, 10mM Tris, 2mM EDTA, 150mM NaCl, pH 7.4) at different time points during 24 hrs post-CHX treatment, and protein levels were analyzed by Western blotting.

11. Cytotoxicity assay

The cytotoxicity was determined using the Roche cytotoxicity detection kit (LDH) (Sigma-Aldrich, St. Louis, MO) according to the manufacturer's protocol. Briefly, 50µL of the conditioned medium was mixed with 50µL of the reaction mixture and incubated in dark for 20 min at room temperature. The absorbance at 490 nm was read using the Wallac 1420 Victor2 Multilabel Microplate Reader (PerkinElmer, Waltham, MA).

12. Statistics

Statistical analysis was performed using GraphPad Prism 5 (GraphPad Prism Software inc., La Jolla, CA). To determine the Gaussian distribution of the data and the variance equality, the D'Agostino & Pearson omnibus normality test was applied. One Sample t-test, a standard unpaired Student's t-test, Mann–Whitney U test or one-way factorial ANOVA followed by Bonferroni's post-hoc analysis was applied to compare the data. A p-value of <0.05 was considered a predetermined threshold for statistical significance. All values are given as means ±

SD. The calculation of sample-size was based on previous results from our laboratory and power calculations (Maesako et al., 2017). Briefly, we have used the GFP-PS1-RFP conformation-sensitive FRET biosensor which has approximately 10% dynamic range and needed to have approximately 25 cells/group to reach statistical difference in the spectral FRET analysis. All experiments were repeated in, at least, three independent experiments and the number of biological replicates was shown. The investigators were blinded during data analysis.

References

Komatsu N, Aoki K, Yamada M, Yukinaga H, Fujita Y, Kamioka Y, Matsuda M. (2011) Development of an optimized backbone of FRET biosensors for kinases and GTPases. Mol Biol Cell 22, 4647-4656

Maesako M, Horlacher J, Zoltowska KM, Kastanenka KV, Kara E, Svirsky S, Keller LJ, Li X, Hyman BT, Bacskai BJ, Berezovska O. (2017) Pathogenic PS1 phosphorylation at Ser367. eLife Jan 30;6. pii: e19720.

Mumm JS, Schroeter EH, Saxena MT, Griesemer A, Tian X, Pan DJ, Ray WJ, Kopan R. (2000) A ligand-induced extracellular cleavage regulates gamma-secretase-like proteolytic activation of Notch1. Mol Cell. 5: 197–206

Uemura K, Lill CM, Li X, Peters JA, Ivanov A, Fan Z, DeStrooper B, Bacskai BJ, Hyman BT, Berezovska O. (2009) Allosteric modulation of PS1/gamma-secretase conformation correlates with amyloid beta(42/40) ratio. PloS one 4, e7893

Uemura K, Farner KC, Hashimoto T, Nasser-Ghodsi N, Wolfe MS, Koo EH, Hyman BT, Berezovska O. (2010) Substrate docking to gamma-secretase allows access of gamma-secretase modulators to an allosteric site. Nat Commun Nov 30;1:130
